# Peptide lineup against Gram-negative bacterial infection – first-in-class peptide inhibitor of H. pylori HtrA

**DOI:** 10.1186/1758-2946-6-S1-P46

**Published:** 2014-03-11

**Authors:** Anna M Perna, Thomas Schmidt, Tim Fugmann, Nicole Tegtmeyer, Steffen Backert, Silja Wessler, G Schneider

**Affiliations:** 1 Department of Chemistry and Applied Biosciences, Swiss Federal Institute of Technology (ETH), Zurich, 8093, Switzerland; 2 Department of Molecular Biology, University of Salzburg, Salzburg, 5020, Austria; 3 Philochem AG, Otelfingen, 8112, Switzerland; 4 Department of Biology, University of Erlangen-Nuremberg, Erlangen, 91058, Germany

## 

More than 50% of the world population is infected with *Helicobacter pylori* (*H. pylori*) and the actual *H. pylori* treatments fail with increased regularity because of continuously rising antibiotic resistances. To meet this challenge, we focus on the development of a new anti-infective therapy against *H. pylori* by targeting a secreted enzyme, high temperature requirement A (HtrA). Release of the serine protease HtrA near the host’s gastric epithelial cells leads to loss of cellular adhesion due to E- cadherin cleavage [1]. We investigated the substrate cleavage sites of HtrA in its natural substrate E-cadherin performing a label-free mass spectrometry-based proteomic analysis and identified preferred cleavage positions by Edman sequencing. Further, we developed the first HtrA peptide inhibitor by synthesizing cleavage site fragments and analogues. Surface plasmon resonance (SPR) was used to perform binding studies. *In vitro* substrate cleavage assays as well as cellular infection assays fully support the biophysical data.
